# Transplantation of Defined Populations of Differentiated Human Neural Stem Cell Progeny

**DOI:** 10.1038/srep23579

**Published:** 2016-03-31

**Authors:** Jeff M. Fortin, Hassan Azari, Tong Zheng, Roya P. Darioosh, Michael E. Schmoll, Vinata Vedam-Mai, Loic P. Deleyrolle, Brent A. Reynolds

**Affiliations:** 1 Department of Neurosurgery, McKnight Brain Institute, University of Florida, Gainesville, FL 32610-0261, USA; 2 Neural Stem Cell and Regenerative Neuroscience Laboratory, Department of Anatomical Sciences &Shiraz Stem Cell Institute, Shiraz University of Medical Sciences, Shiraz, Iran

## Abstract

Many neurological injuries are likely too extensive for the limited repair capacity of endogenous neural stem cells (NSCs). An alternative is to isolate NSCs from a donor, and expand them *in vitro* as transplantation material. Numerous groups have already transplanted neural stem and precursor cells. A caveat to this approach is the undefined phenotypic distribution of the donor cells, which has three principle drawbacks: (1) Stem-like cells retain the capacity to proliferate *in vivo*. (2) There is little control over the cells’ terminal differentiation, e.g., a graft intended to replace neurons might choose a predominantly glial fate. (3) There is limited ability of researchers to alter the combination of cell types in pursuit of a precise treatment. We demonstrate a procedure for differentiating human neural precursor cells (hNPCs) *in vitro*, followed by isolation of the neuronal progeny. We transplanted undifferentiated hNPCs or a defined concentration of hNPC-derived neurons into mice, then compared these two groups with regard to their survival, proliferation and phenotypic fate. We present evidence suggesting that *in vitro*-differentiated-and-purified neurons survive as well *in vivo* as their undifferentiated progenitors, and undergo less proliferation and less astrocytic differentiation. We also describe techniques for optimizing low-temperature cell preservation and portability.

Neurological diseases today afflict about a billion people, accounting for 12% of human mortalities worldwide, and the incidence is expected to rise with an aging population^1^. The existence of stem cells in the adult mammalian^2^, particularly adult human^3^ central nervous system (CNS) makes it feasible for neurological injuries to undergo repair by endogenous mechanisms. Unfortunately, adult neurogenesis is likely not robust enough to address the severity of many injuries^4^,^5^. As another option, neural stem cells (NSCs) and precursor cells (NPCs) can be harvested from a donor, and then expanded in tissue culture for the purpose of later transplantation. Indeed, neural cell replacement therapy is a promising method to help regenerate the afflicted CNS, and the promise of this approach has inspired enormous amounts of global research. In light of the numerous types of neurodegenerative diseases and neurological insults diagnosed increasingly on an annual basis, it would seem that these research efforts are well placed.

NSCs and NPCs have been transplanted as heterogeneous, undifferentiated material by many research groups, in animal models as well as clinically^1^,^4^,^6^,^7^. A caveat to this approach is the undefined phenotypic distribution of the donor cells, which has three principle drawbacks: (1) Stem-like cells retain the capacity to proliferate deleteriously within the host^8^,^9^. (2) There is little control over the donor cells’ terminal differentiation, e.g., a graft intended to replace lost neurons might choose a predominantly glial fate^10^,^11^,^12^,^13^,^14^. (3) There is insufficient ability of researchers to manage and modulate the specific combination of terminal cell types in pursuit of a precise injury treatment (i.e., there is limited investigative power). Controlling the terminal phenotypic fate of grafted cells has long been a challenge in the field. NSCs and NPCs implanted into the CNS have primarily become astrocytes^10^,^11^,^12^,^13^,^14^, which are inadequate by themselves to constitute neural networks and can even have adverse effects such as allodynia^10^,^15^. Shortcomings such as these have inspired many groups to innovate ways of manipulating donor cells *in vitro*, prior to transplant, with the aim of enhancing transplant precision and functional outcome^16^,^17^,^18^. Here we demonstrate a procedure for differentiating human neural precursor cells (hNPCs) in tissue culture, followed by isolation of the neuronal progeny from the glia. We reasoned that by providing heterogeneous hNPCs with neuronal-inducing factors *in vitro*, and then subjecting the differentiated cells to a phenotype-based separation technique before grafting, a high yield of donor astrocytes would be avoided. We hypothesized that transplanting a high concentration of immature neurons into the host CNS would result in fewer surviving donor astrocytes, as compared to transplanting a heterogeneous population of undifferentiated hNPCs. The phenotypic predictability of such a pre-defined, neuronally-enriched human donor cell graft after a prolonged period *in vivo* has not been directly investigated. In multiple experiments in this study, either undifferentiated hNPCs, or a defined concentration of hNPC-derived immature neurons were transplanted into immune-compromised mice. The two graft types were compared with regard to their *in vivo* survival, proliferative capacity and phenotypic fate. We present evidence suggesting that pre-differentiated, purified grafted cells survive as well *in vivo* as their heterogeneous, undifferentiated progenitors, and undergo less proliferation and less astrocytic differentiation. We also demonstrate accompanying procedures for improved hNPC low-temperature preservation and portability, vitally necessary components in “off-the-shelf” cell-based strategies of replacing tissue lost to injury or disease.

## Methods

### Cell maintenance

hNPCs were harvested from the telencephalon of a single fetus at 10 weeks of age, after routine legal abortion, as published previously^19^,^20^,^21^. For transducing the hNPCs, the lentiviral vector encoding enhanced green fluorescent protein (eGFP) was produced as described previously^22^.

hNPCs were cultured in the neurosphere assay (NSA)^2^,^23^,^24^,^25^, as a non-adherent culture, supplemented with factors to encourage growth and proliferation, while suppressing differentiation. Briefly, for the NSA, single cells were plated free-floating, at 100,000 cells/ml in serum-free NS-A medium (90% Human Neurocult NS-A Basal Medium plus 10% Human NeuroCult NS-A Proliferation Supplements, #05750 and 05753, respectively; StemCell Technologies, Vancouver, BC, Canada), supplemented with recombinant human epidermal growth factor (#236-EG; R&D Systems, Minneapolis, MN, USA) at a working concentration of 20 ng/ml, recombinant human basic fibroblast growth factor (#233-FB/CF; R&D Systems) at a working concentration of 20 ng/ml, heparin (#H3149; Sigma-Aldrich, St. Louis, MO, USA) at a working concentration of 0.7 USP units/ml, recombinant human leukemia inhibitory factor (#LIF1050; Millipore, Darmstadt, Germany) at a working concentration of 10 ng/ml, and dehydroepiandrosterone (#A8500-000; Steraloids Inc, Newport, RI, USA) at a working concentration of 1 μM, in untreated tissue culture flasks (Nunc, Waltham, MA, USA). The cells were routinely cultured in a 37 ^o^C and 5% CO_2_ incubator. The culture medium volume was raised by 30% after every 5 days in the feeding regimen. A standard protocol was used for passaging the hNPCs^26^, in which the neurospheres were collected and pelleted (centrifugation at 100 G for 5 min) every 10–12 days. The pellet was re-suspended in 0.05% Trypsin (0.53 mM EDTA) for about 2 min at 37 °C. Soybean trypsin inhibitor was then added, with gentle trituration, to help dissociate the neurospheres into single cells. hNPCs were then re-pelleted, to remove the trypsin and inhibitor mixture.

### Cell differentiation

hNPCs were differentiated towards a neuronal fate using the neuroblast assay (NBA), a protocol we first described using mouse cells^26^,^27^,^28^,^29^. First, hNPCs were plated as single cells at very high density (120,000–160,000 cells/cm^2^), as adherent monolayers in flasks (Nunc, Waltham, MA, USA) pre-coated with poly-ornithine (the flasks had been pre-coated overnight with a solution of 1.5 parts 0.01% poly-ornithine (Sigma) and 8.5 parts 1X sterile PBS, and washed three times with 1X sterile PBS (15 min per wash)). The NBA medium was serum-free, devoid of growth factors, and supplemented with 20 ng/ml brain derived neurotrophic factor (BDNF) (R&D CAT#248-BD) and 1 μg/ml laminin (23017–015; Invitrogen, Waltham, MA, USA). Immature neurons and astrocytes were harvested after 6 days. Prior work in our laboratory has indicated that this hNPC line does not yield a notable amount of oligodendrocytes, except under the appropriate conditions (e.g., with PDGF supplementation) (data not shown). The neurons and astrocytes were dislodged by adding 0.05% Trypsin (0.53 mM EDTA), and incubating at 37 °C for 2 min. Soybean trypsin inhibitor was added to stop trypsin enzymatic activity, followed by mechanical detachment of cells from the flask by firm tapping. The cell pellet was collected by centrifugation at 100 G for 5 min, and the supernatant containing trypsin and inhibitor mixture was removed.

### Cell purification

Magnetically activated cell sorting (MACS), an immunomagnetic cell purification technique, was utilized. Upon NBA harvest, immature neurons and astrocytes were incubated with the immature-neuron-specific antibody, anti-PSA-NCAM, conjugated to phycoerythrin (PE). A PE selection cocktail solution was applied, which bound to the conjugated PE, following which dextrin-coated magnetic nanoparticles were added to the solution. The PE selection construct has affinity for dextrin, resulting in a complete complex. A vial containing the solution and cells was fitted into a magnetic encasement and, over a 5-minute period, the magnetized cells were drawn to the sides of the vial. While still within the magnetic encasement, the vial was turned upside down, allowing the solution to escape. Only the PSA-NCAM-negative astrocytes escaped with the dispensed liquid.

### Cell cooling procedure

To test the feasibility of shipping hNPCs on ice (4 °C), hNPCs were placed either as single cells or neurospheres in a 4 °C refrigerator, in 2 ml of regular cell culture media (described above). After 2 or 4 days, hNPCs were removed from the refrigerator and returned to normal culturing conditions in the 37 °C and 5% CO_2_ incubator. After a recovery period of one week, fold expansion data collection began for a minimum of 3 cell passages per thawed cell vial source, and a minimum of two thawed cell vial sources per experimental group.

### Cell cryopreservation procedure

Conditions for freezing the hNPC-derived, MACS-purified immature neurons involved two comparative cryoprotective groups; the conventional 10% DMSO in regular cell culture media (described above) and the animal component-free Cryo-Store 10 from BioLife Solutions, Inc. Cell solutions of both groups were placed in cryovials (8 cryovials per group), which were placed into a Thermo Scientific Mr. Frosty freezing container with 100% isopropyl alcohol. The Mr. Frosty was then placed in −80 °C for various durations, ranging from 1–60 days. Viability was measured upon thaw using trypan blue (TB) uptake using a hemocytometer, as well as by propidium iodide (PI) uptake using flow cytometry. Survival frequency was calculated by dividing total TB-negative or PI-negative cells by the total number of cells.

### Immunocytochemistry

The cell solution (200 cells/μL) was treated with laminin (described above). 70 μL of this cell solution was added to each well of a 384 well plate (Nunc, Waltham, MA, USA) (the plate was pre-coated with poly-ornithine (described above). The plate was incubated at 37 °C overnight, before the media was replaced with cold 4% formaldehyde for a 15 min fixation at 4 °C. The wells were then emptied and refilled with 1X sterile PBS and stored at 4 °C.

For immunostaining, cells were first blocked in a solution of 1X PBS with 0.1–0.3% Triton X-100 and 5% normal goat serum, and then incubated at 4 °C overnight in primary antibody cocktails containing the following, as needed: rabbit anti-GFAP (1:750; catalog #Z0334; Dako), rabbit anti-Ki67 (1:1200; NCL-Ki67p; Novocastra), rabbit anti-Doublecortin (1:600; ab77450; Abcam). Cells were washed in 1X PBS and then incubated with secondary antibody (Alexa 594; A-11012; Invitrogen) diluted 1:700 in 1X PBS. After washes, cells were stained in 4′,-6′ diamidino-2-phenylindole (DAPI) (Molecular Probes, 1:1000) for 5 min and washed.

### Transplantation procedures

All animal protocols were approved by the Institutional Animal Care and Use Committee (IACUC) at the University of Florida (UF), and the methods were carried out in accordance with the approved guidelines.

#### Neonatal mouse transplantation procedure

Neonatal NOD-*scid* gamma (NSG) mice were grafted at postnatal day zero (P0) to maximize host developmental signaling, which has been shown to assist donor cells^30^. NSG pups were anaesthetized on ice for approximately 15 minutes. The right lateral ventricle (LV) was targeted by first locating bregma and then by injecting about 1 mm lateral to bregma (approximately 2 mm depth). Using a Stoelting syringe micromanipulator (Stoelting Company, Wood Dale, IL), a 33-gauge needle was lowered into the pup’s head, penetrating the soft skull. The cells (either GFP-expressing hNPCs or GFP-expressing immature neurons) were injected over a 3-minute period. The total injection volume was 1 μL of 100,000 cells/μL in 1X PBS. The injected pups were kept warm and revived, by placing on a rodent warming device for approximately 15 minutes before returning to their cage. The mice in this experiment were allowed to survive either 10 days or 8 weeks after transplantation.

#### Adult mouse transplantation procedure

NOD-*scid* mice were grafted at 3 months of age. Mice were anaesthetized with isoflurane and placed in a Stoelting stereotactic frame (Stoelting Company, Wood Dale, IL). A 12 mm skin incision was first made, exposing the skull. The right striatum was targeted by first locating bregma and then injecting at the following coordinates (a burr hole was initially drilled into the skull): A/P 0 mm, M/L 2.7 mm, D/V 1 mm. A 33-gauge needle (Hamilton Company, Reno, NV) delivered the cells (either GFP-expressing hNPCs or GFP-expressing MACS-purified immature neurons), as a 1 μL injection of 300,000 cells/μL in 1X PBS. Cells were injected over a 4 min period. The incision was then sutured. The mice were kept warm and revived from anaesthesia, by placing them on a rodent warming device for approximately 15 minutes before returning to their cage. The mice in this experiment were allowed to survive 6 months after transplantation.

### Tissue processing and immunohistochemistry

Mice were sacrificed after the indicated duration post transplant, via deep anaesthetization with isoflurane followed by transcardial perfusion with 4% formaldehyde in 1X PBS, pH 7.4. Tissue was post-fixed in 8% formaldehyde in 1X PBS, pH 7.4, at 4 °C overnight, and then cryoprotected in 30% sucrose in 1X PBS, pH 7.4, at 4 °C for 2 days. For frozen sections, brains were frozen in optimal cutting temperature compound (OCT™) and sectioned in the sagittal plane at 30 μm thickness. For immunostaining, free-floating sections were blocked in a solution of 1X PBS with 0.1–0.3% Triton X-100 and 5% normal goat serum, and then incubated at 4 °C overnight in primary antibody cocktails containing the following, as needed: rabbit anti-GFAP (1:500; catalog #Z0334; Dako), rabbit anti-Ki67 (1:500; NCL-Ki67p; Novocastra), rabbit anti-Doublecortin (1:1,000; ab77450; Abcam). Sections were washed (3 × 15 minute washes) in 1X PBS and then incubated with fluorophore-conjugated secondary antibody (Alexa 594; A-11012; Invitrogen) diluted 1:500 in 1X PBS. After washing the secondary stain away once with 1X PBS, sections were stained in 4′,-6′ diamidino-2-phenylindole (DAPI) (Molecular Probes, 1:1000) for 5 min and then washed again (3 × 15 minute washes).

### Quantification of donor cell survival

Quantification of donor cell survival was performed by an investigator blinded to the cell transplantation groups. First, fluorescent images of all brain areas containing GFP+ cells were taken at 10X and 20X magnification (Leica DMI 4000 B). The images were imported into ImageJ software, and the total number of nucleated GFP+ cells were counted in each image. For each brain, every 30 μm thick section of a complete 1-in-6 serial series, spanning the entire brain, was used for calculating the number of nucleated GFP+ cells per section.

### Quantification of donor cell phenotypes

A spinning disk confocal microscope (Olympus) was utilized to image a minimum of 50 nucleated GFP+ events per brain, for each phenotypic marker. Each image was captured as a series of 1 μm-thick planes, to enable 3-dimensional scrutiny using ImageJ software. GFP+ cells were classified as neurons or astrocytes if co-labeled with DCX or GFAP, respectively.

## Results

### Cryo-banked hNPCs represent a renewable, portable source of cells

**Figure 1:**
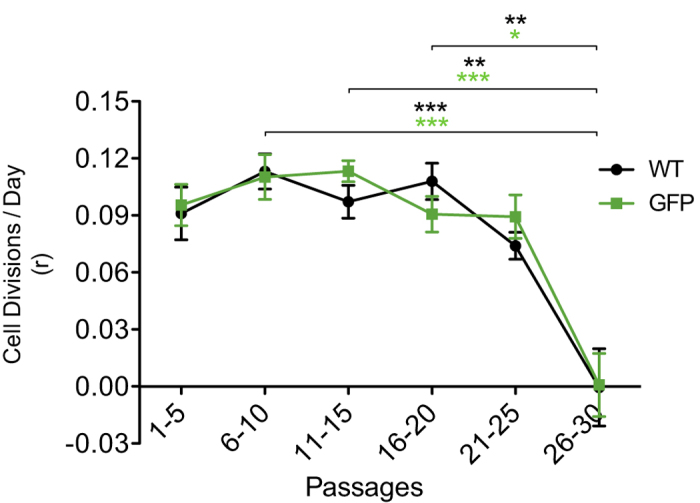
*In vitro* fold expansion across passages. hNPCs remained stably able to proliferate, despite passaging tweny-five times over nearly a one-year timespan. Transducing hNPCs with GFP did not affect proliferative performance of these cells compared to WT. hNPC serial passage data permitted the calculation of a rate constant (r), or cell divisions/day, using an exponential equation. Means + s.e.m. *P < 0.05, **P < 0.01, ***P < 0.001, by one-way ANOVA, followed by Bonferroni’s test.

Of importance in the neural cell replacement field is the standardization of stable, renewable cell lines^31^. Therefore, it is essential that such cell lines be well characterized with respect to their self-renewal capacity at baseline and over extended passages in tissue culture. We measured hNPC expansion rate over nearly a 1-year timespan (thirty 12-day-long passages, each passage being 10–12 days). The cells exhibited a consistent expansion rate of about 3–4 fold per passage, during the first 21–25 passages. To control against anyconfounding influence by GFP on cell proliferation, GFP-transduced cells were analyzed under the same protocol as wild type (WT) cells. No significant difference was observed between GFP and WT, relating to fold expansion, over as long as 1 year *in vitro* (Fig. 1). It is also noteworthy that the x-axis on the graph refers to passages post-thaw, and not passages post-dissection (i.e., hNPCs in actuality experienced about twenty passages prior to being cryopreserved as a large series of vials, from which one vial of GFP and one vial of WT were thawed to begin the experiment). Therefore, results demonstrate that these hNPCs are reliable as a long-term renewable source of neural tissue, whether or not they express GFP. In transplant biology, the utility of GFP as a reporter is increased when its function does not intrude on other aspects of an experiment. In this regard, our data suggests that GFP does not interfere with cell maintenance and viability.

**Figure 2:**
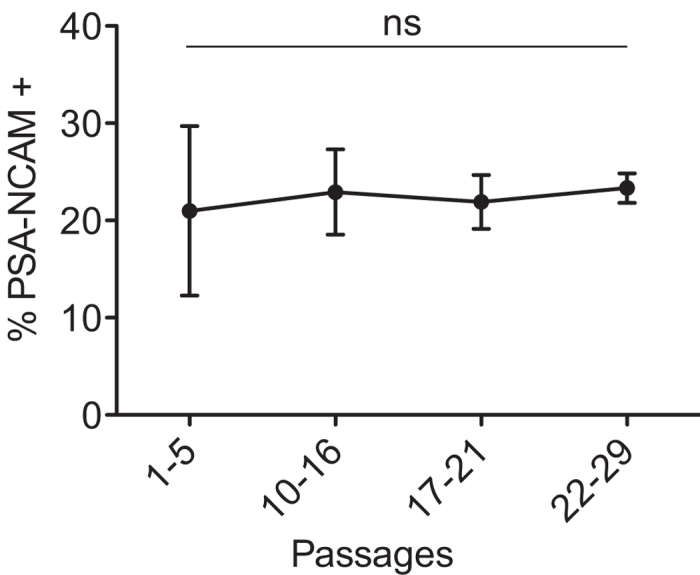
*In vitro* neurogenicity across passages. hNPCs remained stably able to differentiate into PSA-NCAM+ neuroblasts, despite passaging tweny-nine times over nearly a one-year timespan. Not significant (ns). Means + s.e.m. *P < 0.05, by one-way ANOVA, followed by Bonferroni’s test.

It is also imperative that a cell line be well characterized as to its multipotency over extended passages in tissue culture. hNPC neuron-forming capacity was measured over nearly a 1-year timespan. Across twenty-nine passages, the cells were periodically tested with respect to their neurogenicity, by plating them in our *in vitro* induced-neurogenesis assay, which we call the neuroblast assay (NBA)^26^,^28^,^32^, to encourage a neural lineage. After 6 days in the NBA, cells underwent magnetically activated cell sorting (MACS) to isolate the neurons from the astrocytes on the basis of PSA-NCAM immunoreactivity. The number of PSA-NCAM+ cells yielded, divided by the number of hNPCs originally plated in the NBA, represented %PSA-NCAM+. Data within each passage group (e.g., passages 1–5) were combined into a single data point. hNPCs remained stably able to differentiate into PSA-NCAM+ neuroblasts, despite passaging tweny-nine times (Fig. 2). This result, along with the data demonstrating continual proliferation *in vitro*, collectively manifest that hNPCs remain healthy, renewable and neurogenic in long-term culture.

**Figure 3:**
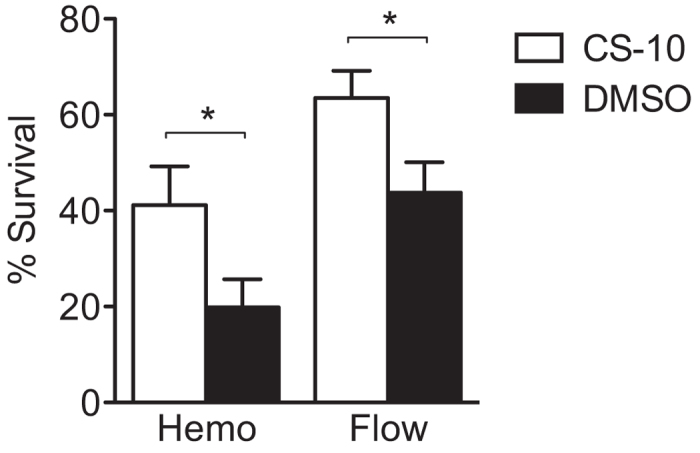
Comparative viability of purified hNPC-derived immature neurons after cryopreserving by conventional 10% DMSO versus CryoStor CS-10. Immature neurons were more viably cryopreserved by CS-10 than by conventional 10% DMSO. Cell-containing vials were kept at −80 °C for various durations, ranging from 1 week to 6 months (data from all time points were pooled). Cell survival was measured by trypan blue uptake using a hemocytometer, as well as by propidium iodide uptake using flow cytometry. Means + s.e.m. *P < 0.05, by unpaired two-tailed *t* test.

To optimize cell preservation and storage processes, multiple methods were assessed. Purified hNPC-derived immature neurons were experimentally cryopreserved in two different cryopreservative solutions, CS-10 from StemCell Technologies and the conventional mixture of media with 10% DMSO. Cells from each group were thawed at the same time and immediately quantified for uptake of trypan blue and propidium iodide, via hemocytometry and flow cytometry, respectively. Immature neurons frozen with CS-10 survived significantly better than those frozen with 10% DMSO/media (Fig. 3). This result suggests that CS-10 is a superior reagent for cryopreserving hNPC progeny.

**Figure 4:**
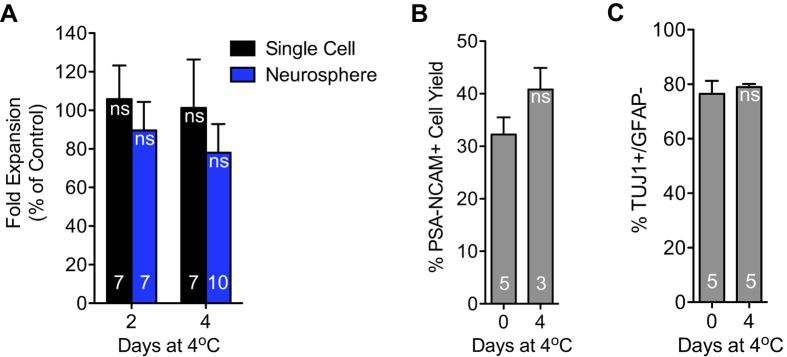
Characterization of hNPCs subjected to 2–4 days at 4 °C. (**A**) Fold expansion rate of cells serially passaged (a minimum of 4 passages) after 2–4 days incubation at 4 ^o^C was not significantly affected compared to control. Cells incubated as single cells proliferated the same amount as cells incubated as un-dissociated neurospheres. (**B,C**) hNPCs incubated at 4 ^o^C performed similarly to control when differentiated for six days in the neuroblast assay, regarding number of PSA-NCAM+ cells yielded per originally plated (**B**) and proportion of TUJ1+/GFAP− cells amongst the unpurified yield (**C**). Means + s.e.m. *P < 0.05, *ns* means not significant, by unpaired two-tailed *t* test. The numbers on bars indicate the numbers of cell passages or number of neuroblast assays, including two different thawed cell vial sources.

We also sought to verify that temporary cooled conditions would not alter the viability of the cells. hNPCs were stored for 2 days and 4 days in a 4 °C refrigerator unit, enclosed in falcon tubes, as single cells or as neurospheres. The cells were then returned to culture and their viability was assayed via fold expansion rate over multiple passages. hNPCs could indeed be stored at 4 °C for as long as 4 days and still proliferate on par with control cells (Fig. 4A). No significant difference has yet been observed between storing cells at 4 °C as single cells versus neurospheres. Furthermore, we found that hNPC neurogenicity had been maintained, despite incubating at 4 °C for 4 days (Fig. 4B,C). This may validate the practice of shipping undifferentiated cells at 4 °C, as a practical method of avoiding the considerable time and attention required to freeze-thaw hNPCs. From these data, it is evident that successful inter-facility transport of hNPCs can be achieved by simply cooling the cells to hypothermia, and hence suppressing their level of metabolism^33^, while avoiding the cumbersome and damaging process of freezing the cells.

### Differentiated hNPC neural and glial progeny can be purified and recombined in controlled distributions

**Figure 5:**
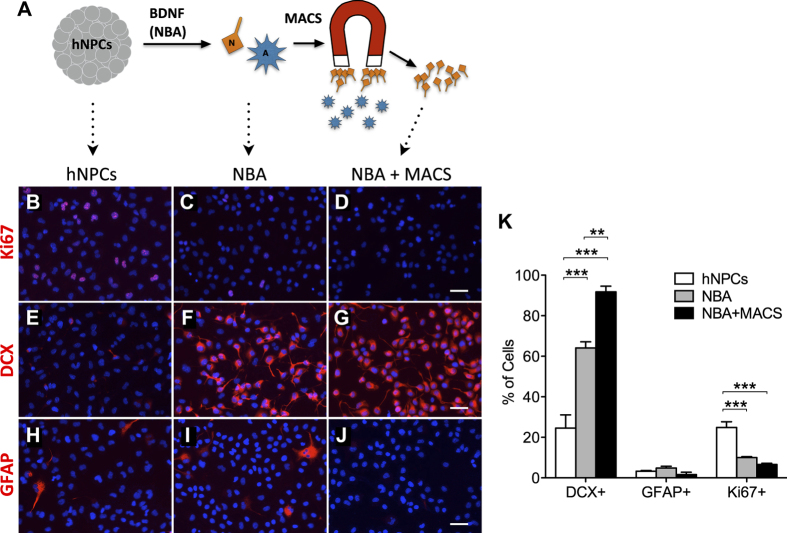
*In vitro* phenotypes of hNPCs differentiated in the neuroblast assay (NBA), followed by enrichment of the immature neuron subpopulation using MACS. (**A**) Overall experimental design. hNPC neurospheres were dissociated into single cells and plated in the NBA, generating a mixed population of neurons (N) and astrocytes (**A**). The neurons were then isolated from the astrocytes via MACS. Lastly, phenotyping was performed on the cells during the three major phases of the experiment. (**B–D**) Fluorescent images of immunocytochemical staining show hNPCs significantly lose expression of the proliferation marker Ki67 during the NBA. Enrichment of the neuron subgroup after the NBA, using MACS, reduces Ki67 expression still further (scale bar = 20 μm) (49,6-diamidino-2-phenylindole (DAPI), blue). (**E–G**) hNPCs significantly gain expression of the immature neuron marker DCX during the NBA. Enrichment of the neuron subgroup after the NBA yields an even higher percentage of DCX+ cells (scale bar = 20 μm) (49,6-diamidino-2-phenylindole (DAPI), blue). (**H–J**) Expression of the astrocyte marker GFAP increases slightly during differentiation, but MACS purification removes virtually all the percentage of GFAP+ cells (scale bar = 20 μm) (49,6-diamidino-2-phenylindole (DAPI), blue). (**K**) Quantification of immunolabeled cells indicates the differentiation of hNPCs into immature neurons during the NBA, and the subsequent purification of neurons made possible by MACS. Means + s.e.m. *P < 0.05, **P < 0.01, ***P < 0.001, by one-way ANOVA, followed by Bonferroni’s test.

We argue that it will benefit the field of cell therapy to apply techniques for pre-defining not only the phenotypic lineage of donor cells, but also the relative distribution of their phenotypes. Therefore, hNPCs were cultured in the NBA, followed by cell purification using MACS to isolate an enriched population of neurons. The cells were also immunolabeled at each stage, i.e., at hNPC, post-NBA and post-MACS stages (Fig. 5A–J). The percentage of cells expressing DCX, GFAP and Ki67 was then counted (Fig. 5K). As expected, as hNPCs were first cultured under differentiative conditions and then purified for neuronal content, their expression of DCX continually rose, while Ki67 continually declined (Fig. 5K). GFAP is apparently upregulated by 1.5% during the NBA (Fig. 5K), despite supplementation with BDNF. This can likely be understood as a result of growth factor withdrawal and the strong gliogenic predisposition of some cells. However, MACS is able to remove the astrocytic cells very effectively. Thus, hNPC-derived immature neurons can be robustly generated using the NBA, and the neurons can be efficiently isolated from glia using MACS.

**Figure 6:**
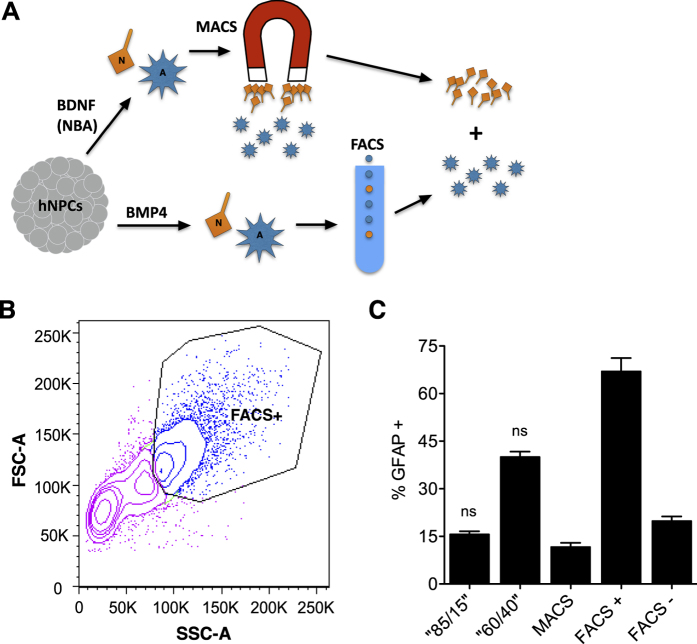
*In vitro* distribution of GFAP+ hNPC progeny after incrementally mixing BMP4-generated astrocytes with NBA-generated, MACS-purified neurons. (**A**) Overall experimental design. hNPCs were plated in either the NBA or an alternative, astrocyte-generating assay using BMP4. MACS was used to generate a purified population of neurons (N), while FACS was used to generate a purified population of astrocytes (**A**). Lastly, purified neurons and astrocytes were re-combined in defined percentages. (**B**) FACS gating strategy to enrich the BMP4-induced hNPC progeny for astrocytes, using SSC vs. FSC to select the largest 25% of cells (FACS+). (**C**) Quantification of GFAP-immunolabeled cells indicates that the targeted cell phenotype distributions (85% neurons: 15% astrocytes, 60% neurons: 40% astrocytes) were achieved (no statistically significant difference between actual and targeted values). The MACS column represents the purified neuron population, while the FACS+ and FACS− columns represent the larger (targeted for selection) and smaller (not targeted for selection) cohorts of the BMP4-generated cells, respectively. Not significant (ns). Means + s.e.m. *P < 0.05, by unpaired two-tailed *t* test.

Grafting pre-determined ratios of cells may allay the risks of tumorigenesis and too many unwanted cell phenotypes. Furthermore, this approach will allow for cell dosing studies, by precisely modulating the distribution of cell types we engraft. To demonstrate that controlled ratios of neurons to glia could be formulated *in vitro*, which could hypothetically be used for grafting in the CNS, we generated, purified and then recombined neuron and astrocyte populations derived from hNPCs (Fig. 6A). To do this, differentiated and purified astrocytes were needed to mix with our MACS-yielded neurons. Hence, hNPCs were first cultured for 6 days in growth factor-withdrawn media inoculated with bone morphogenetic protein (BMP4). As BMP4 is an astrocyte differentiation factor^34^, this assay was intended to generate a significantly higher number of GFAP+ cells than the NBA. The harvested cell population was then purified for astrocytes by selecting the largest 25% of total cells, utilizing fluorescence-activated cell sorting (FACS) (Fig. 6B). Next, the MACS-purified neurons were mixed with the FACS-purified astrocytes, with the aim of attaining defined neuron:astrocyte populations of 85:15 and 60:40. Lastly, the final mixtures were immunostained with anti-GFAP to quantitatively verify that the targeted phenotype ratios had been achieved. In fact, the “85:15” and “60:40” groups each consisted of very near their targeted percentage of astrocytes (16% and 40% GFAP+ cells, respectively) (Fig. 6C). The presence of a low number of GFAP+ cells amongst the physically smaller FACS- cells is not surprising. Under differentiative conditions, immature neural cells can exist for a period as “hybrid asterons,” where they may possess morphologic and phenotypic characteristics that are a mixture between neurons and astrocytes^35^. Likely, most of these cells will eventually become astrocytes. The overall result of this experiment provides proof of principle and suggests that hNPC-derived purified neurons can be recombined with hNPC-derived purified astrocytes to produce controlled distributions of cell phenotypes, for further *in vitro* studies or for grafting.

### Grafted purified neurons survive as well, and generate fewer astrocytes, than their hNPC progenitors

**Figure 7:**
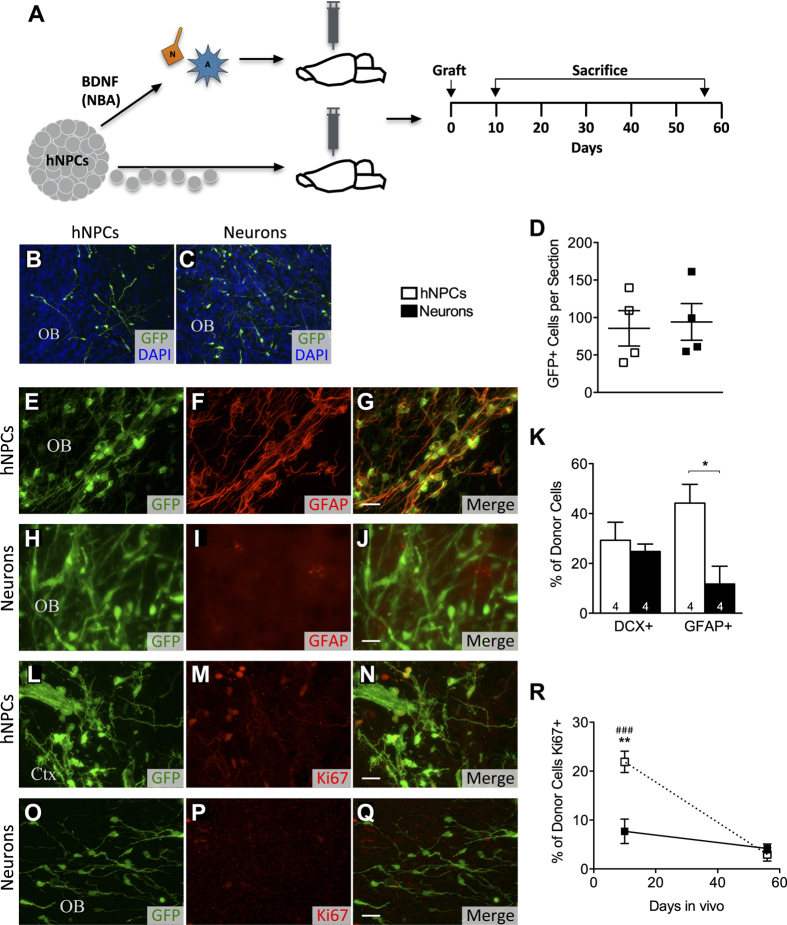
*In vivo* survival and phenotypes of hNPCs and their neuronal progeny transplanted in P-zero NSG mice. (**A)** Overall experimental design. One group of mice was grafted with hNPCs, while the other group was grafted with NBA-generated hNPC progeny. Mice in both groups were sacrificed after 10 and 56 days. (**B,C**) Representative images of GFP+ surviving cells in mice grafted with either hNPCs (**B**) or immature neurons (**C**) 8 weeks after transplantation. (**D**) Quantification of surviving GFP+ cells in mice grafted with either hNPCs sacrificed 8 weeks after transplantation. The number of surviving GFP+ cells per section were not significantly different between hNPC and neuron grafts. (**E–J**) Fluorescent images of immunohistochemical staining, 8 weeks after transplantation, show GFP+/GFAP+ hNPCs to be upregulated in the hNPC (**E–G**) versus neuron (**H–J**) grafts (scale bar = 20 μm). (**K**) Quantification of donor cell phenotypes in the ipsilateral ventricle, cortex, hippocampus, RMS, olfactory bulb and cerebellum. While no difference in DCX frequency was seen between grafted cell groups 8 weeks after transplantation, the hNPC graft did yield significantly more GFP+/GFAP+ cells. (**L–R**) Likewise GFP+/Ki67+ cells are more observable in hNPC (**L–N**) versus neuron (**O**–**Q**) grafts, 10 days after transplantation (scale bar = 20 μm). Quantitatively, (**R**) GFP+/Ki67+ cells were more frequent in the hNPC grafts than in the neuron grafts 10 days after transplantation (**), but this difference gradually disappeared by 8 weeks post implant. (OB = olfactory bulb, Ctx = cortex) Means + s.e.m. ^###^*P* < 0.001 (hNPCs at 10 days versus 8 weeks), *P < 0.05, **P < 0.01, by unpaired two-tailed *t* test.

In another published study, our same line of hNPCs was grafted into the parietal cortex of neonatal mice. After 8 weeks *in vivo*, the hNPCs differentiated into functional GABAergic interneurons and glutamatergic pyramidal neurons, capable of receiving both excitatory and inhibitory inputs^36^. In the present study, two cell groups were compared. hNPC-derived immature neurons (directly harvested from the NBA) and hNPCs were intracranially grafted in NSG mice at post-natal day 0, into the right lateral ventricle (Fig. 7A). After 10 days, some donor cells in both transplant groups remained in clumps in the ventricle and along the needle injection tract in the cortex, while many cells had begun to exit the ventricle along the rostral migratory stream (RMS). Some animals of both groups also displayed donor cells in the ipsilateral olfactory bulb. After 56 days, both implanted cell groups had ceased to remain in clumps and had healthy splayed-out appearances with migratory, sinuous processes, in virtually all animals (Fig. 7B,C). Many donor cells were present in the ipsilateral ventricle, cortex, hippocampus, RMS, olfactory bulb and cerebellum. No significant difference in donor cell survival was observed between the transplant groups (Fig. 7D). Thus, hNPC-derived immature neurons can survive as well as hNPCs 8 weeks after implanting in uninjured, neonatal mice.

We next classified surviving donor cells as immature neurons or astrocytes 8 weeks after transplantation, using DCX and GFAP, respectively. Donor astrocytes often result from grafts of multipotent neural tissue, either due to the superior hardiness of astrocytes over neurons, or perhaps due to the host milieu providing cues for cells to choose an astrocytic fate. However, immunohistochemical staining shows GFP+/GFAP+ cells to be more frequent in the hNPC versus neuron grafts (Fig. 7E–K). No difference in DCX frequency was seen between groups (Fig. 7K). Thus, grafted hNPC-derived immature neurons generate fewer astrocytes than hNPCs 8 weeks after implanting in uninjured neonatal mice.

We next classified surviving donor cells as proliferative or non-proliferative in the host during the 8 weeks following transplantation. Neither donor cell group exhibited signs of tumor formation throughout the entire experiment. At 10 days, Ki67 expression was significantly higher in the hNPC than neuron grafts (Fig. 7L–R). While this difference did level off by 56 days (Fig. 7R), the initial presence of proliferative donor cells may relate to actual graft-derived tumorigenesis seen in other studies^8^,^9^. The concerning fact is that, with the hNPC graft, proliferating cells were implanted; and proliferating cells are less predictable as to their fate. With heterogenous, multipotent grafts, the pool of cells with proliferative potential is greater than with pre-differentiated grafts, even though the proliferation rate measured at 56 days in this study’s experimental conditions do not show a difference between the groups; more permissive conditions may allow these proliferative properties to be expressed.

**Figure 8:**
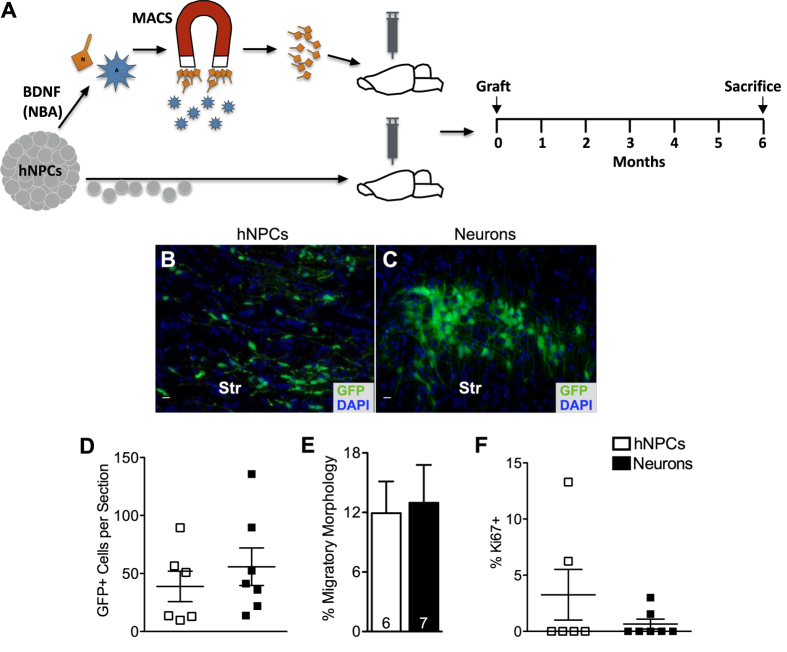
*In vivo* potential tumorigenesis assay design, as well as percent survival and Ki67 expression of late-passage hNPCs and their neuronal progeny. (**A**) Experimental timeline, wherein 3 month old NOD-*scid* mice were grafted with either hNPCs or MACS-purified neurons (N) in the striatum, and allowed to survive for 6 months. (**B,C**) Representative images of GFP+ surviving cells in mice 6 months after grafting with either hNPCs (scale bar = 10 μm) (**B**) or immature neurons (scale bar = 10 μm) (**C**). (**D**) Difference in overall donor cell survival between cell groups is not significant. (**E**) Migration of implanted cells was estimated on the basis of “migratory profiles characterized by fusiform-shaped cell bodies with single leading and/or trailing processes” (Zheng *et al.*^38^). For both cell groups, the total number of GFP+ cells displaying a migratory morphology was counted per the total number of GFP+ cells. Both cell groups displayed migratory characteristics. (**F**) Proliferative status of both cell groups is very low, as indicated by Ki67 immuno-reactivity, particularly in the neuron group. (Ctx = cortex, Str = striatum) Means + s.e.m. *P* > 0.05, unpaired two-tailed *t* test.

In a separate transplant experiment, we directly addressed another challenge in cell replacement therapy, to limit the risk of tumorigenesis by grafted tissue. We proposed that an approach to doing this is to pre-define the maturity and phenotypes of the donor cells, rather than to indiscriminately implant cells that potentially contain subpopulations which retain stem-like characteristics. We reasoned that hNPCs might be considered an uncontrolled dose, whereas the MACS-purified neuronal progeny might be considered defined and less tumorigenic. To test this hypothesis, hNPCs vs. MACS-purified immature neurons were grafted into the striatum of adult NOD-*scid* mice, which were sacrificed after 6 months (Fig. 8A). High passage number cells were used to conduct this experiment, reasoning that genomic instability would be increasingly more likely to beset a line of cells the longer they were cultured *in vitro*^37^. Additionally, a relatively high number of cells were implanted so as to increase the likelihood of tumorigenesis within the timespan of this experiment. However, after 6 months, none of the grafted animals exhibited signs of tumor formation. Histology of the harvested brains showed surviving donor cells for both groups (Fig. 8B,C), with the purified neuron graft and the hNPC graft surviving comparably, yet overall graft survival was low (Fig. 8D). Migration of implanted cells was estimated on the basis of “migratory profiles characterized by fusiform-shaped cell bodies with single leading and/or trailing processes,” as described in Zheng *et al.*^38^. Both cell groups displayed migratory characteristics (Fig. 8E). We stained the brain tissue with Ki67 to assess the proliferative character of the donor cells. Low overall Ki67 expression by the grafted cells was found in both groups (Fig. 8F). These combined data suggest that hNPC-derived purified neurons survive as well as hNPCs 6 months after implanting in healthy adult mice. Although data from the majority of mice in this experiment suggests that neither group poses a significantly greater turmorigenic threat than the other, it should be noted that the *in vivo* duration and number of animals tested here are miniscule in comparison to the duration and scale of anticipated clinical applications. Furthermore,two animals in the hNPC group harbored donor cells with high Ki67 expression (13.3 and 6.3%), which may foretell a dangerous level of proliferation.

## Discussion

In neural cell replacement therapy, the need for a renewable source of cells has been evident since the controversial early days of fetal neural tissue grafts for Parkinson’s disease patients^39^. As renewable human cell sources are recently becoming available^40^,^41^,^42^,^43^,^44^,^45^, there is now the need to ensure reliability of these cell sources as they begin to be cultured^46^,^47^. Long-term culture of eventual donor tissue, in turn, highlights the importance of having reliable cell cryopreservation and portability techniques^48^. Additionally, a prevalent challenge upon grafting undifferentiated cells has been that donor tissue in the host environment may differentiate into undesired phenotypes^10^,^11^,^15^, or proliferate uncontrollably^8^,^9^,^49^,^50^. Furthermore, the very nature of implanting mixed precursors also necessitates a concomitant inability for researchers to modulate the grafted cellular dose to fit a particular application. Given the obstacle that the field has encountered of ensuring that enough donor cells survive to achieve efficacy^47^,^51^, it seems all the more vital that the pre-grafted distribution of cell phenotypes be optimized^16^. We argue that the future of cell-based strategies to treat the damaged nervous system could benefit from transplanting defined cell populations. This study demonstrates methods for potentially addressing each of the above issues, reporting a reliable and neurogenic, renewable source of cells, improved cryopreservation and portability techniques, as well as efficient differentiation and purification assays (a means of creating any desired cellular distribution of glia-to-neurons).

To demonstrate the long-term *in vitro* renewability and neurogenicity of hNPCs, we revived hNPCs from cryopreservation and cultured them for about a year. Both GFP-transduced and non-transduced hNPCs were observed to be consistently self-renewing for almost nine months, before proliferation rates began to decline (possibly related to the stress of handling or a reduction in the number of bona fide stem cells in the population). With many vials of hNPCs frozen at the same low passage number, one vial at a time can be thawed up and placed in culture to be used for several months of experiments. To further evaluate the long-term reliability of the hNPCs *in vitro*, we measured their immature neuron-forming capacity across twenty-nine passages. It was important to characterize the neurogenicity of the hNPCs, since we aimed eventually to graft defined populations of their differentiated progeny. It was confirmed that the hNPCs could consistently generate PSA-NCAM+ neuronal cells during nearly a year of passaging, at a steady frequency of about 22%. This means that the average number of PSA-NCAM+ cells that can be routinely isolated from a single culture round is about 22% of the initial number of hNPCs plated in the NBA.

This study was primarily designed to address the challenges of grafted cell fate and survival by pre-differentiating hNPCs *in vitro* and then transplanting a defined cell population. We sought to determine whether we would be able to generate more neurons and fewer astrocytes if a population that was pre-enriched for hNPC progeny, which had chosen a neuronal lineage, were implanted. We sought to answer this question in the naïve rodent brain environment, aiming to provide the field with proof of principle that transplant material can be engineered *in vitro,* as regards phenotypic distribution and maturity, and that such engineering can lead to predictable *in vivo* donor cell characteristics. It has been argued that a potential pitfall of transplanting post-mitotic cells, which are therefore arguably less hardy, is the risk of low donor cell survival^51^,^52^. However, if the intention of a transplant procedure is, for example, to replace dead or dying neurons, then grafting pure neurons is potentially more efficacious than grafting a low percentage of neurons, even provided that the latter graft survives twice as well. Using a high percentage of immature human neurons harvested from the NBA, we found that these neuronal cells actually survive as well as their undifferentiated progenitors upon intraparenchymal grafting in naïve mice. We also rightly predicted that the purified neuronal graft would bring about fewer astrocytes *in vivo* than the undifferentiated heterogeneous graft, because the former cells were first reared *in vitro* for 6 days under conditions favoring neurogenesis, reducing the cells’ phenotypic flexibility. Since fewer glia were yielded, it means that the shortcomings of excess astrocytes, such graft inefficacy or even allodynia, may be avoided through the approach outlined herein. Yielding fewer glia, and more neurons, is also important for applications in which neuronal cell architecture specifically needs to be replaced, such as in the chronic stages of ischemic stroke^53^, or supplemented as in temporal lobe epilepsy^54^,^55^. Conversely, for treating more acute injury environments, where an astrocyte graft may secrete trophic factors and scavenge glutamate and free radicals^56^,^57^, the differentiation and purification methodologies demonstrated in this study would still hold promise. Intriguingly, DCX expression was not significantly different between transplant groups. We had predicted that the purified neuronal graft would yield more neurons, and in turn fewer astrocytes, than the undifferentiated heterogeneous graft, yet, surprisingly, only the later prediction proved accurate in this study. At the time of grafting, the neuron group was not 100% neurons but rather about 65% neurons and 2% glia (3 times more DCX+ cells than in the hNPC group, while GFAP+ cell frequency was comparable to the hNPC group). The immature neuron graft could possibly have shifted toward an astrocytic character, so-called “phenotypic fluidity”^35^, though too immature to express GFAP. Alternatively, the neuron graft may have shifted in its neuronal character, such as to cease expressing DCX and begin expressing a different, maybe more mature, neuronal marker. While further characterizational research is required, we postulate that the neuron graft underwent some combination of these scenarios.

Certainly safety is the first priority in clinical cell therapy, and transplanting uncommitted stem and precursor cells, such as NPCs, has raised legitimate concerns regarding the potential for long-term donor cell tumorigenicity^8^,^9^,^49^,^50^. This study asked the question if implanting a purified population of human immature neurons, versus their mixed hNPC progenitors, would reduce tumorigenic risk. We sought to address this by subjecting NBA-harvested cells to MACS and implanting the yielded, purified population of immature neurons into adult NOD-*scid* mice and allowing the animals to survive for 6 months post implant. Purified neurons and mixed hNPC groups survived similarly and performed safely in this study. While the data was variable and not statistically significant, there was indeed more Ki67 expression in the hNPC graft, particularly by two mice within the group, suggesting that the hNPCs might reveal a significantly greater tumor risk than the purified neurons if tested on a larger scale, i.e., if more animals were grafted, and with a higher number of cells per animal, and sacrificed after a greater duration post transplant. While the results seen in this study favorably indicate that transplanting mixed hNPCs is as safe as transplanting their post-mitotic progeny, further studies are necessary to truly allay concerns about the long-term behavior of potentially proliferative donor tissue.

As reported in their 2007 paper, Foroni *et al.* propagated adult mouse NSCs for more than a year *in vitro* (over 120 passages), under mitogenic culture conditions similar to those in the present study. They analyzed the NSCs at early, middle and late passages. During this time, the expression profile of an array of genes, which had previously been shown to correlate with malignant phenotypes, did not exhibit any significant change. Furthermore, karyotype analysis was performed in the same study, leading to the conclusion that aberrant cell transformation had not occurred during extensive culturing^58^. In the present study, we demonstrated the performance stability of the cultured hNPCs; specifically their consistent growth rate and neurogenicity. Consistent with our results are the findings of other studies that have demonstrated long-term functional stability and self-renewal of cultured human CNS stem cells^59^,^60^. Again, these groups utilized culture conditions similar to the ones we used in this study. It is also of note that we grafted fifteen mice with the hNPC cell line and observed no tumor formation even after 6 months. The nature of the existing concern over donor cell genetic stability is that, while they may show functional and phenotypic stability *in vitro*, the cells may all the while undergo genetic changes that do not manifest until exposure to the *in vivo* environment. Therefore, the results of the potential tumorigenesis assay reported herein provide supportive evidence for the genetic stability of this particular cell line. The safety of grafting cell lines in the clinic, however, will certainly benefit from screening for genetic transformations with the utmost caution.

This study demonstrates the long-term maintenance, cryopreservation and portability of a renewable line of human fetal neural cells, as well as methods for the differentiation and then purification of the immature human neurons. This technology of *in vitro* cell differentiation and purification may be useful for studies in drug screening, neurotoxicology and electrophysiology, in addition to precisely treating CNS disorders. The transplant paradigm presented here has potential to reduce the hazards of uncontrolled donor cell phenotypic fates, such as tumorigenesis^8^ or predominant astrocytic differentiation^10^,^15^. However, whether or not the survival of desired cell types is enhanced remains questionable. While this technique has been demonstrated herein to allow for grafting a more pure population of cells, future work is necessary to confirm that the technique enhances *in vivo* survival of specified cell types. As cell therapy moves more and more into the clinic, this kind of precision will be imperative. This is a method by which researchers can study the impact of different, uniquely defined cell doses on very specific injury environments, e.g., acute vs. chronic injury stages. The investigative power afforded by grafting defined cell populations may help the field to tease out answers needed to contribute to the mission of raising neural transplant biology to a level of consistent benefit to public health.

## Additional Information

**How to cite this article**: Fortin, J. M. *et al.* Transplantation of Defined Populations of Differentiated Human Neural Stem Cell Progeny. *Sci. Rep.* **6**, 23579; doi: 10.1038/srep23579 (2016).
